# Efficacy of Information-Motivation-Behavioral Skills ModelBased nursing care intervention in management of patients with Heart Failure: A retrospective cohort study

**DOI:** 10.12669/pjms.40.11.10750

**Published:** 2024-12

**Authors:** Yinxia Luo, Xinmei Qi, Oushan Tang

**Affiliations:** 1 Yinxia Luo, Department of Cardiovascular Internal Medicine, Shaoxing Second Hospital Medical Community General Hospital, Shaoxing, Zhejiang Province 312000, P.R. China; 2 Xinmei Qi, Department of Cardiovascular Internal Medicine, Shaoxing Second Hospital Medical Community General Hospital, Shaoxing, Zhejiang Province 312000, P.R. China; 3 Oushan Tang, Department of Cardiovascular Internal Medicine, Shaoxing Second Hospital Medical Community General Hospital, Shaoxing, Zhejiang Province 312000, P.R. China

**Keywords:** Heart Failure, Information Motivation Behavioural Skills Model, Nursing

## Abstract

**Objective:**

Self-care practices are integral part of heart failure (HF) management. Information-Motivation-Behavioral skills (IMB) model has shown promise in fostering better self-care behaviors, but its efficacy in management of HF patients remains underexplored. This retrospective study investigated the value of IMB model-based nursing intervention for improving self-care, hemodynamic indicators, and quality of life (QOL) of HF patients.

**Methods:**

This retrospective study involved 308 HF patients treated at a tertiary care hospital from January 2021 to January 2023. Patients were grouped based on the received nursing care. The intervention group contained patients who received IMB model-based nursing intervention (n=151), and a control group included patients who got routine nursing care (n=157). IMB intervention comprised information delivery, motivation enhancement, and behavioral skills training. Key outcomes included self-care behavior measured by the Self-Care of HF Index (SCHFI), quality of life, assessed by the Minnesota Living with HF Questionnaire (MLHFQ), and hemodynamic parameters including Left Ventricular End-Systolic Diameter (LVESD), Left Ventricular End-Diastolic Diameter (LVEDD), and Left Ventricular Ejection Fraction (LVEF).

**Results:**

IMB model-based nursing intervention was significantly more effective in improving the condition of HF patients than the routine nursing care, including better SCHFI and MLHFQ scores and improved hemodynamic parameters (LVESD, LVEDD, and LVEF) (P<0.05). Variation in different analysis further confirmed the efficacy of the IMB model-based nursing intervention.

**Conclusion:**

IMB model-based nursing intervention significantly improved self-care behavior, QOL, and hemodynamic parameters in HF patients. This mode of nursing care has a potential as a valuable strategy for enhancing HF management.

## INTRODUCTION

Heart failure (HF) remains a significant healthcare challenge worldwide.^1^ Currently, HF affects 26 million people worldwide, and the prevalence of the condition is on the rise with the gradual aging and increased survival of patients with antecedent heart disease worldwide.^2^,^3^ Despite current advances in the field, HF still leads to substantial morbidity, mortality, and a healthcare burden.^2^ It is estimated that about half of the patients diagnosed with HF will die within five years, highlighting the severity and poor prognosis associated with this condition.^4^

While medical and surgical treatments play pivotal roles in managing HF, optimal care extends beyond these conventional strategies,^5^ and involves comprehensive, individualized care, including effective self-care practices. Self-care in HF refers to the decisions and actions taken by patients to maintain adequate life, healthy functioning, and well-being while living with the condition.^6^ Self-care measures include medication adherence, symptom monitoring, physical activity, dietary restrictions, and seeking assistance when health status changes. Effective self-care practices in HF patients have been linked to improved clinical outcomes, including fewer hospitalizations, improved quality of life, and lower mortality.^6^

However, numerous studies have revealed that self-care in HF is frequently suboptimal, and a significant proportion of HF patients do not adhere to recommended self-care behaviors.^7^ Various factors, including a lack of information, poor motivation, and behavior-related skills, have been identified as barriers to effective self-care among HF patients.^8^ Information-Motivation-Behavioral skills model (IMB model) is a health behavior change model that has shown promise in addressing these barriers.^9^ It proposes that information relevant to the change of health behavior, personal motivation to modify behavior, and the necessary behavioral skills are all fundamental determinants of health behavior change.^9^ IMB model has been widely used in various health contexts, including HIV/AIDS management, diabetes care, and hypertension control, and has been effective in improving health outcomes.^9^–^14^

In the context of HF, IMB model-based nursing interventions can potentially improve self-care practices by providing disease-specific information, enhancing motivation to adhere to recommended self-care behaviors, and equipping patients with necessary self-care skills. However, while these interventions can potentially optimize health outcomes in HF patients, studies that research the impact of IMB model-based nursing interventions in the context of HF self-care are scarce. Moreover, there is still very limited understanding of the potential effects of IMB model on hemodynamic indicators and overall quality of life of HF patients, which is crucial for evaluating HF management strategies. Therefore, assessing these factors may not only enhance our understanding of the value of IMB model in HF self-care but also contribute to developing more effective nursing interventions. This retrospective study assessed the impact of IMB model-based nursing intervention on self-care, hemodynamic indicators, and QOL of patients with HF compared to the routine nursing care.

## METHODS

This is a retrospective cohort study, aiming to assess the impact of IMB model-based nursing intervention on HF patients. The study population included HF patients aged ≥18 years, who received care between January 2021 and January 2023, at the Heart Failure Center of Shaoxing Second Hospital, a tertiary care hospital.

### Ethical Approval:

The ethics committee of the hospital approved this study with the number: 2022050, Date: May 15^th^ 2022.

### Inclusion Criteria:

The eligibility criteria were as follows:

- a definitive diagnosis of HF based on the European Society of Cardiology (ESC) criteria,^15^
- an ability to communicate effectively,
- a willingness to participate in the study.

### Exclusion Criteria:

- Patients with cognitive impairments,
- Psychiatric disorders, or other severe comorbidities that would affect their capacity to participate

Clinical records of patients were retrospectively grouped according on the treatment they received during their stay at the HF Center. The intervention group was comprised of patients who received IMB model-based nursing intervention, and the control group included patients on routine nursing care. IMB model-based nursing intervention contained three components.

The information component provided patients with HF-related knowledge, covering topics such as disease pathophysiology, prognosis, and importance of self-care. The motivation component was aimed at enhancing the patient’s internal motivation to engage in self-care practices. This was achieved by reinforcing the benefits of self-care and addressing any concerns or fears. The behavioral skills component focused on training patients on necessary self-care skills, including symptom monitoring, medication management, diet regulation, and physical activity.

### Data collection:

Data were retrieved from electronic health records. Patient demographic data included gender, age, and educational level. Clinical characteristics included duration of the disease, cardiac function (per New York Heart Association (NYHA) functional classification), cause of HF, payment method, and presence of comorbid conditions such as hypertension and diabetes. Patient-reported outcomes, including the Self-Care of HF Index (SCHFI) and Minnesota Living with HF Questionnaire (MLHFQ), were also collected.

### Self-Care of HF Index (SCHFI):

The Self-Care of HF Index (SCHFI) is a validated method of assessing self-care behavior in HF patients. This tool comprises three subscales, including self-care maintenance, management, and confidence.^16^ Self-care maintenance subscale evaluates adherence to medications and diet, regular exercise, and symptom monitoring. Self-care management subscale assesses how well patients respond to symptoms when they occur, particularly their ability to recognize changes in symptoms and take appropriate action. Self-care confidence subscale measures the confidence in performing self-care behaviors. Each SCHFI item is rated on a four-point Likert scale. Higher scores are indicative of better self-care behavior. Scores for each subscale are standardized to a 100-point scale. Scores below 70 suggesting inadequate self-care.

### Minnesota Living with HF Questionnaire (MLHFQ):

QOL of patients was evaluated by a self-reported Minnesota Living with HF Questionnaire (MLHFQ) that has been well-validated in multiple languages and various cultural contexts.^17^ MLHFQ comprises 21 items divided into two dimensions: physical (eight items) and emotional (5 items), with an additional 8 items that are not distinctly physical or emotional. QOL is assessed according to the six-point Likert scale, where 0 indicates no effect, and five indicates significant effect. A total score ranges from 0 to 105, and higher scores means poorer QOL. This tool allows patients to express how much their HF symptoms affect their daily life and well-being, thus providing clinicians with insights into the subjective experience of living with HF.

### Echocardiographic parameters:

Key echocardiographic parameters, such as Left Ventricular End-Systolic Diameter (LVESD),^18^ Left Ventricular End-Diastolic Diameter (LVEDD),^19^ and Left Ventricular Ejection Fraction (LVEF),^20^ were measured using standard echocardiographic techniques following the American Society of Echocardiography (ASE) guidelines.^21^

### Statistical Analysis:

Data analysis was done using the STATA software, version 17. Quantitative data were presented either as averages with standard deviation (SD) or as medians and interquartile range (IQR). Distribution normality was assessed by Shapiro-Wilk test. Continuous data were analyzed by independent t-tests (normal distribution), and the Mann-Whitney U test (non- normal distribution). Categorical variables were depicted as proportions and percentages, and intergroup variations were evaluated by chi-square or Fisher’s exact tests, as required. A difference-in-difference analysis was employed across all parameters between both groups to evaluate the influence of the IMB model-based nursing intervention on patient results. P<0.05 was statistically significant.

## RESULTS

As shown in Table-I, median age of patients in the intervention group (n=151) was 67.50 (±9.40) years. Patients in the control group (n=157) were aged 68.37 (±10.06) years in average (p=0.43). Gender, education level, cardiac function, marital status, cause of disease, payment method, hypertension, and diabetes were similar in both groups. Baseline echocardiographic parameters, SCHFI and MLHFQ scores of the intervention and the control groups were comparable (P>0.05). Table-II.

**Table-I:** Baseline parameters of the patients (N=308).

Parameter	Intervention Group (n=151)	Control Group (n=157)	p-value
Age (years)	67.50 (±9.40)	68.37 (±10.06)	0.43
Gender			0.512
- Male	94 (50.54%)	92 (49.46%)	
- Female	57 (46.72%)	65 (53.28%)	
Education			0.169
- Below High School	107 (46.72%)	122 (53.28%)	
- Above High School	44 (55.70%)	35 (44.30%)	
Cardiac Function			0.516
- Grade II	16 (41.03%)	23 (58.97%)	
- Grade III	61 (48.80%)	64 (51.20%)	
- Grade IV	74 (51.39%)	70 (48.61%)	
Marital Status			0.789
- Married	123 (49.40%)	126 (50.60%)	
- Single	28 (47.46%)	31 (52.54%)	
Cause of Disease			0.345
- Coronary Heart	61 (44.53%)	76 (55.47%)	
- Cardiomyopathy	42 (51.22%)	40 (48.78%)	
- Valvular Disease	48 (53.93%)	41 (46.07%)	
Payment Method			0.619
- Insurance	135 (48.56%)	143 (51.44%)	
- Self	16 (53.33%)	14 (46.67%)	
Hypertension			0.114
- No	97 (45.97%)	114 (54.03%)	
- Yes	54 (55.67%)	43 (44.33%)	
Diabetes			0.468
- No	112 (47.86%)	122 (52.14%)	
- Yes	39 (52.70%)	35 (47.30%)	

**Table-II:** Baseline differences in echocardiographic parameters, SCHFI and MLHFQ scores between intervention and control group (N=308).

Parameters	Control Group (Mean ± SD) (n=157)	Intervention Group (Mean ± SD) (n=151)	P-value
***Echocardiographic parameters***			
LVESD (in mm)	54.03 ± 4.51	53.40 ± 4.48	0.2215
LVEDD (in mm)	60.76 ± 3.97	61.32 ± 3.63	0.1925
LVEF (%)	44.92 ± 3.42	45.57 ± 3.17	0.0834
***Self-Care of HF Index (SCHFI)***			
Self-care maintenance	39.64 ± 11.13	38.80 ± 11.16	0.5081
Self-care management	36.59 ± 9.34	35.93 ± 10.61	0.5670
Self-care confidence	38.71 ± 9.55	39.23 ± 10.33	0.6478
***Minnesota Living with HF Questionnaire (MLHFQ)***			
Body	39.45 ± 5.82	40.21 ± 6.00	0.2648
Emotion	52.25 ± 8.22	52.86 ± 7.81	0.5034
Others	54.99 ± 8.59	55.73 ± 8.47	0.4507

*All values are rounded to two decimal places. P-value is for the difference between the means of the intervention and control groups, where a p-value of less than 0.05 would be statistically significant. LVESD - Left Ventricular End-Systolic Diameter; LVEDD - Left Ventricular End-Diastolic Diameter; LVEF - Left Ventricular Ejection Fraction.

After the nursing intervention, LVESD parameters of the intervention group (44.56 ± (4.33mm) were significantly lower compared to the control group (49.24 ± 4.36mm) (p<0.001). Similarly, post-intervention LVEDD of the intervention group was significantly lower (55.33 ± 3.72mm *vs* 57.77 ± 3.95mm in the control group, p<0.001), and post-intervention LVEF values were significantly higher (58.56 ± 3.35% vs 49.94 ± 3.64% in the control group, p<0.001) compared to patients who received standard nursing care. IMB model-based nursing intervention was also linked to a marked improvement in SCHFI and MLHFQ scores (Table-III; p<0.001).

**Table-III:** Post-intervention differences in echocardiographic parameters, SCHFI and MLHFQ scores between intervention and control group (N=308).

Parameters	Control Group (Mean ± SD) (n=157)	Intervention Group (Mean ± SD) (n=151)	P-value
***Echocardiographic parameters***			
LVESD (in mm)	49.24 ± 4.36	44.56 ± 4.33	<0.001
LVEDD (in mm)	57.77 ± 3.95	55.33 ± 3.72	<0.001
LVEF (%)	49.94 ± 3.64	58.56 ± 3.35	<0.001
***Self-Care of HF Index (SCHFI)***			
Self-care maintenance	42.28 ± 10.67	52.07 ± 9.96	<0.001
Self-care management	38.94 ± 8.96	50.28 ± 10.29	<0.001
Self-care confidence	41.86 ± 9.83	49.89 ± 10.69	<0.001
***Minnesota Living with HF Questionnaire (MLHFQ)***			
Body	56.74 ± 6.37	62.33 ± 7.84	<0.001
Emotion	63.18 ± 8.43	73.94 ± 8.03	<0.001
Others	63.98 ± 8.87	74.51 ± 9.04	<0.001

*All values are rounded to two decimal places. P-value is for the difference between the means of the intervention and control groups, where a p-value of less than 0.05 would be statistically significant. LVESD - Left Ventricular End-Systolic Diameter; LVEDD - Left Ventricular End-Diastolic Diameter; LVEF - Left Ventricular Ejection Fraction.

Variation in different estimations, comparing IMB model and standard care, showed that IMB model was associated with significant improvements in LVESD (Diff-in-Diff estimate: -4.058, p<0.001), LVEDD (Diff-in-Diff estimate: -3.006, p<0.001), and LVEF (Diff-in-Diff estimate: 7.955, p<0.001) post-intervention. Additionally, SCHFI and MLHFQ scores were also markedly improved in the intervention group of patients. Table-IV.

**Table-IV:** Difference-in-difference estimation results comparing the IMB model against standard care for the study parameters.

Outcome Variable	Difference-in-Difference Estimate	Standard Error	t-value	P-value
***Echocardiographic parameters***				
LVESD (in mm)	-4.058	0.713	5.69	<0.001
LVEDD (in mm)	-3.006	0.616	4.88	<0.001
LVEF (%)	7.955	0.548	14.51	<0.001
***Self-Care of HF Index (SCHFI)***				
Self-care maintenance	10.635	1.732	6.14	<0.001
Self-care management	11.988	1.582	7.58	<0.001
Self-care confidence	7.509	1.628	4.61	<0.001
***Minnesota Living with HF Questionnaire (MLHFQ)***				
Body	4.833	1.056	4.58	<0.001
Emotion	10.150	1.310	7.75	<0.001
Others	9.794	1.410	6.95	<0.001

Subgroup analysis revealed that the improvement in SCHFI scores was more pronounced among female patients compared to male patients (Mean difference: 8.67 vs. 5.89, p<0.05). Similarly, patients aged 70 years and above showed a greater enhancement in MLHFQ scores (Mean difference: 12.34, p<0.01) compared to younger patients, indicating that the IMB model-based nursing intervention may be particularly beneficial in older populations and females.

## DISCUSSION

Our findings demonstrated that IMB model-based nursing care was associated with significant improvements in both clinical outcomes (echocardiographic parameters) and patient-reported outcomes (SCHFI and MLHFQ) compared to routine nursing care. It further showed considerable improvement in both self-care behaviors and the QOL of HF patients that received IMB model-based care. Effective self-care practices have been linked to improved clinical outcomes, including fewer hospitalizations, better QOL, and lower mortality among HF patients.^7^ Significant improvement in SCHFI scores in the intervention group suggests that the IMB model-based nursing successfully enhanced self-care practices among these patients. This underscores the crucial role of nursing interventions not just in the provision of care but also in the empowerment of patients towards better self-care.

The substantial improvement in MLHFQ scores in the intervention group points to an enhanced quality of life post-intervention. HF is known to severely impact the quality of life due to burdensome symptoms, repeated hospitalizations, and the required complex self-care regimen.^13^ Our finding that the IMB model-based intervention effectively improved the QOL is encouraging, suggesting the potential of this approach to address the challenges associated with living with HF. This study has showed that IMB mode-based nursing leads to more significant improvements in echocardiographic parameters, including LVESD, LVEDD, and LVEF compared to the routine nursing care. Our observations suggest that the implementation of the IMB model in nursing care for HF patients does not only enhance self-care and quality of life but can potentially favorably modulate cardiac structural and functional parameters. As these parameters directly impact morbidity and mortality in HF, this improvement could potentially lead to a reduction in adverse clinical outcomes.

However, further research is needed to validate this potential effect. The decrease in LVESD and LVEDD post-intervention suggests reduced left ventricular dilation. As left ventricular dilation often occurs in HF, and is associated with poor prognosis,^22^ our results are indicative of improved HF status of patients who received IMB mode-based care. Similarly, the increase in LVEF, a key index of left ventricular systolic function, aligns with improved heart function, as reduction in LVEF is commonly observed in HF with reduced ejection fraction.^23^

The observed improvements in self-care behavior, QOL, and hemodynamic parameters suggest that the IMB model-based nursing intervention operates on multiple layers to enhance HF management.^24^ Information component of the model may increase patients’ understanding of their condition, emphasizing the importance of self-care in managing HF, and likely leading to better adherence to self-care practices. Improved self-care, in turn, can lead to better management of HF symptoms, less physical distress, and a resultant improvement in the quality of life. Moreover, better self-care can potentially lead to optimal control of cardiac load and stress, thereby leading to improvements in cardiac function and structure. Motivation component could have enhanced the patients’ internal motivation to engage in self-care, positively influencing self-care behaviors. The behavioral skills component might have equipped patients with necessary self-care skills, enabling them to manage their condition more effectively.

### Limitation:

This is a retrospective study which could lead to potential biases, such as selection and information bias. IMB intervention was provided by different nursing professionals, and individual differences in the delivery of the intervention could potentially influence the outcomes. However, the consistency of the observed effects across different outcomes suggests that the intervention itself rather than individual professionals’ execution might have driven the effects. Finally, this is a single-center study which may impact its generalizability. Future studies involving multiple centers and diverse populations are warranted to validate these findings and enhance their generalizability.

Nevertheless, our study offers promising evidence supporting the value of IMB model-based nursing intervention in HF management. This comprehensive approach that addresses informational needs of patients, as well as motivational aspects, and behavioral skills aligns with the holistic care required for chronic conditions like HF. The observed benefits of the IMB model-based intervention reiterate the value of personalized, patient-centered care in HF management. It underscores the role of patients as active participants in their care rather than passive recipients of medical treatments. It also emphasizes the importance of addressing psychological and behavioral aspects, alongside biological aspects, in chronic disease management.

While the study sheds light on new directions for enhancing HF care, it also opens several avenues for future research. Longitudinal studies are warranted to investigate the long-term effects of the IMB model-based intervention on clinical outcomes in HF patients. Future research should also focus on understanding the mechanisms through which the IMB model-based intervention modulates cardiac function and structure. Such insights can not only enhance our understanding of the intervention’s effects but also contribute to its further refinement.

## CONCLUSION

IMB model-based nursing intervention significantly improved self-care behavior, QOL, and hemodynamic parameters in HF patients. This mode of nursing care has a potential as a valuable strategy for enhancing HF management. However, future longitudinal research are warranted to investigate the long-term effects of the IMB model-based intervention on clinical outcomes in HF patients.

### Authors’ contributions:

**YL:** Conceived and designed the study, prepared the manuscript.

**XQ** and **OT:** Collected the data and performed the analysis and critical review.

All authors have read and approved the final manuscript and are responsible for the integrity of the study.
